# Spatial transcriptome reveals histology-correlated immune signature learnt by deep learning attention mechanism on H&E-stained images for ovarian cancer prognosis

**DOI:** 10.1186/s12967-024-06007-8

**Published:** 2025-01-24

**Authors:** Chun Wai Ng, Kwong-Kwok Wong, Barrett C. Lawson, Sammy Ferri-Borgogno, Samuel C. Mok

**Affiliations:** 1 Department of Gynecologic Oncology and Reproductive Medicine, Unit 1362, The University of Texas MD Anderson Cancer Center, 1515 Holcombe Blvd, Houston, TX 77030 USA; 2 Department of Anatomical Pathology, The University of Texas MD Anderson Cancer Center, Houston, TX 77030 USA

**Keywords:** Attention, Deep learning, H&E, Immune signature, Ovarian cancer, Prognosis, Spatial transcriptome

## Abstract

**Background:**

The ability to predict the prognosis of patients with ovarian cancer can greatly improve disease management. However, the knowledge on the mechanism of the prediction is limited. We sought to deconvolute the attention feature learnt by a deep learning convolutional neural networks trained with whole-slide images (WSIs) of hematoxylin-and-eosin (H&E)–stained tumor samples using spatial transcriptomic data.

**Methods:**

In this study, 773 WSIs of H&E-stained tumor sections from 335 patients with treatment naïve high-grade serous ovarian cancer who were included in The Cancer Genome Atlas (TCGA) Pan-Cancer study were used to train, and validate, and to test a ResNet101 CNN model modified with attention mechanism. WSIs from patients in an independent cohort were used to further evaluate the model.

**Results:**

The prognostic value of the predicted H&E-based survival scores from the trained model on patient survival was evaluated. The attention signals learnt by the model were then examined their correlation with immune signatures using spatial transcriptome. After validating the model with the testing datasets, pathway enrichment analysis showed that the H&E—based survival score significantly correlated with certain immune signatures and this was validated spatially using spatial transcriptome data generated from ovarian cancer FFPE samples by correlating the selected signature and attention signal.

**Conclusions:**

In conclusion, attention mechanism might be useful to identify regions for their specific immune activities. This could guide future pathological study for the useful immunological features that are important in modulating the prognosis of ovarian cancer patients.

**Supplementary Information:**

The online version contains supplementary material available at 10.1186/s12967-024-06007-8.

## Background

Advanced ovarian cancer, which has a 5-year survival rate of less than 30%, is the deadliest among gynecologic cancers. Most ovarian cancer is high-grade, serous ovarian cancer (HGSOC), and the poor survival rate among patients with the disease is mainly due to the fact that the disease is usually diagnosed at a late stage [1, 2]. The standard treatment for HGSOC is cytoreductive surgery before platinum-based chemotherapy or after neoadjuvant chemotherapy [3]. However, patient treatment response and survival duration decrease with ages and disease stages [4–6]. In addition, most of the HGSOC patients relapse because they develop resistance to taxane-based chemotherapy. Nevertheless, 15% of patients diagnosed with advanced HGSOC have overall survival (OS) durations of more than 10 years despite the development of recurrent diseases. Discovering predictive markers for prognosis could help researchers to identify therapeutic targets for the disease, thus improving patient survival rates and disease management.

Models that can predict survival in patients with advanced HGSOC have been described recently [7–11], but they have limited performance and usually require transcriptomic analysis or tedious image processing, which can be time consuming and costly, especially in regions with limited resources. Thus, a cost-effective and interpretable method for the prediction of ovarian cancer survival is urgently needed for both patients and clinicians. Such a method would improve treatment decision-making and disease management, especially for patients with elevated risks of poor outcomes [12].

Recent advancements in machine-learning algorithms for computer vision have created an interest in their applicability in digital pathology [13, 14]. Deep learning models trained on data such as histological and computed tomography images have been used to predict signaling activity, mutation and prognosis [15–18]. Although ovarian tumor histological images have been employed to predict patient prognosis, the mechanism of the prediction is not fully understood. The understanding the features learned by the model would enhance the confidence in applying image-based predictive model in clinical setting, and also aid the pathological research of the disease in the future.

In this study, WSIs of H&E-stained tumor sections and clinical data from The Cancer Genome Atlas (TCGA) ovarian cancer dataset were employed to train a model that can be used to predict the survival of HGSOC patients. Validation studies using WSIs of H&E-stained tumor sections obtained from an independent patient cohort from The University of Texas MD Anderson Cancer Center (MDACC) were also performed to further determine the performance of the predictive model. The immunological signatures correlated with the model were determined and validated using spatial transcriptome data of ovarian cancer FFPE samples.

## Methods

### Image and clinical data preparation for model training and validation

WSIs of H&E-stained tumor sections from treatment naïve advanced HGSOC in the TCGA-OV dataset were downloaded from the Genomic Data Commons (GDC) portal using GDC client [19]. TCGA’s clinical data for patients with HGSOC were downloaded from the GDC portal and from the cBioportal (TCGA-OV Pan-Cancer) dataset (Accessed on October 1, 2023) [20, 21]. The cBioportal and GDC data were matched using TCGA patient IDs and then merged. Images from patients of all age and stage III-IV (i.e., images from patients of stage I-II were excluded) were included for training. All the patients were female. The characteristics and demographics of the included patients are shown in Table 1. The median OS and Progression-free survival (PFS) durations were 35 and 15 months, respectively. Most patients were white, and the median age at diagnosis was 60. Only patients with clinical stage III or IV disease were selected. The patients without this information were classified as having stage III disease so that they could be included in this study.

**Table 1:** The clinical characteristics of the patients included in the TCGA dataset

Variable	N = 335
Overall survival in months, median	35
Progression-free survival in months, median	15
Race, n	
American Indian or Alaskan Native	3
Asian	6
Black or African American	19
Native Hawaiian or other Pacific Islander	1
White	301
Not available	5
Age at diagnosis in years, median	60
Residual disease, n	
No Macroscopic disease	39
1–10 mm	171
11–20 mm	24
> 20 mm	73
Not available	28
Clinical stage, n	
I	0
II	0
III	267
IV	68

TCGA, The Cancer Genome Atlas

### Model training with the fivefold cross-validation process and testing

**Fig. 1:**
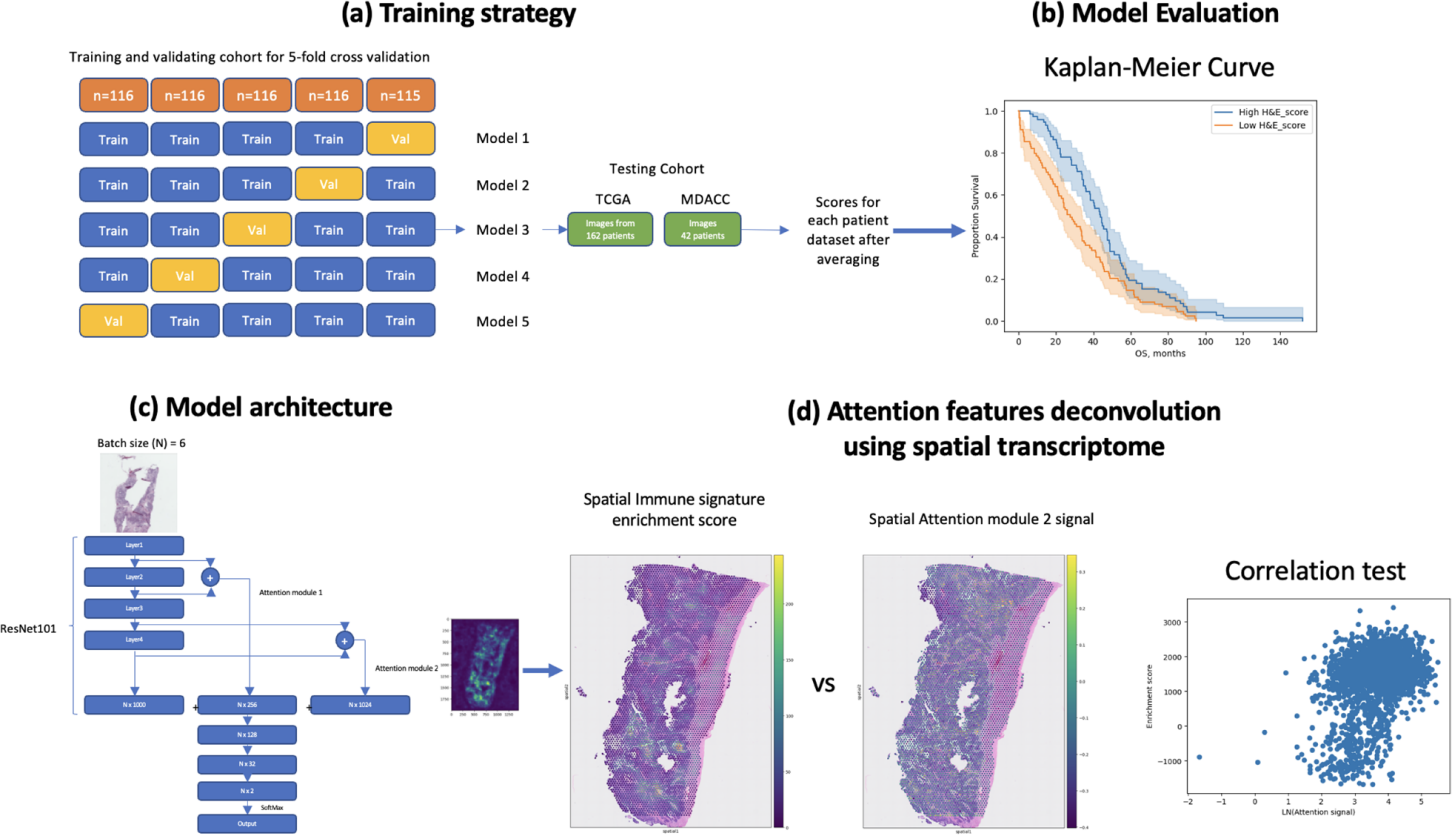
The study overviews

TCGA images included in this study were first separated into training and validation (n = 579), and testing (n = 194) datasets (Fig. 1a). The training and validation dataset was then separated into 5 folds for training (n = 463/464) and validation (n = 116/115) [22]. Models for each fold were trained 5 times for each of the 10 epochs to select the model with the best area under the receiver operating characteristic (AUROC) values, which were then evaluated. After the top models in each fold were selected, the models were then evaluated with the blind testing TCGA and MDACC datasets (Fig. 1b). WSIs of H&E-stained treatment-naïve tumor sections and clinicopathological characteristics from the MDACC dataset were obtained from the ovarian cancer repository of the Department of Gynecologic Oncology and Reproductive Medicine under protocols approved by the University of Texas MD Anderson’s Institutional Review Board. Written informed consent from the patients were obtained by front desk personnel, and the studies were conducted in accordance with recognized ethical guidelines. TCGA data were obtained from public repository and did not require ethical approval.

The fivefold cross-validation process of model training was described (a). The training images were separated into 5 training and validation sets, each with different validation images. From each fold, a model was selected with the best AUROC evaluated using validation images. The models trained were then evaluated with datasets from the testing cohort by averaging the score outputs from the 5 models using Kaplan–Meier curve and log-rank test (b). Training and prediction were performed by feeding images (1024 × 1024 resolution; batch size [N] = 6) into the pretrained ResNet101 CNN model for training (c). The output of layers 1 and 2 of the pretrained ResNet101 model was extracted, as was the output of layers 3 and 4, and the resultant information was used as the attention mechanism. The output of the ResNet101 model was concatenated with the output of attention modules 1 and 2, and was followed by 3 additional layers of fully connected neural networks for a final output (N × 2) after SoftMax processing. The attention features generated by attention module 2 were investigated for their correlation with immune signature enrichment score (d). Abbreviations: MDACC, The University of Texas MD Anderson Cancer Center; TCGA, The Cancer Genome Atlas; Train, training; Val, validation.

### The architecture of the deep learning model with an attention mechanism

The deep learning network was constructed with the PyTorch [23] framework in Python. An overview of the model is shown in Fig. 1c. The input training batch size (N) was 6 images. The Layers 1 and 2 of the pretrained ResNet101 model from PyTorch were interpolated, as were layers 3 and 4. The interpolated layers from the 2 attention modules were processed with AvgPool2d to create a flattened layer (N × 256 and N × 1024, respectively) and were fed into the fully connected neural network layers together with the output of the model (N × 1000) for attention mechanism. The N × 2280 layer was followed by 3 fully connected layers with 128, 32, and 2 perceptrons, respectively. Each of the layers (except the final layer) was followed by a dropout with rate of 0.2 and was activated with a rectified linear unit. The final layer was processed with SoftMax to produce the final output. The losses of between the target and predicted value during training were determined using FocalLoss, and the Adam algorithm was used as an optimizer. The learning rate was 0.0002, and the learning-rate decay was 0.1 for every 7 epochs.

### Spatial immune signature enrichment analysis

The predicted H&E—based survival scores of the images of the TCGA testing cohort were separated into 2 groups (low and high score) by step-wise experiment for the lowest log-rank p-value. The 2 groups were then determined for statistics for the MSigDB c7 immune signatures by GSEA. The most significant signature was selected for the correlation study with the attention signal using spatial transcriptome data of ovarian cancer FFPE samples (Fig. 1d) (https://www.10xgenomics.com/datasets?query=&page=1&configure%5BhitsPerPage%5D=50&configure%5BmaxValuesPerFacet%5D=1000&refinementList%5Bspecies%5D%5B0%5D=Human&refinementList%5BanatomicalEntities%5D%5B0%5D=Ovary&refinementList%5Bplatform%5D%5B0%5D=Visium%20Spatial&refinementList%5BpreservationMethods%5D%5B0%5D=FFPE, accessed on October 1, 2023).

### Image processing and augmentation

The original images were divided into 2 square images (resolution, 1024 × 1024) if their initial widths and lengths were not the same (Supplementary Fig S1a). Both images were fed into model training, whereas only 1 of the images was used for validation and testing. During training, the order of the images was shuffled, normalized, augmented with vertical or horizontal flipping, rotated, and affined randomly. Two examples of image augmentation are shown in Supplementary Fig S1b.

### Statistical methods

The AUROC was determined using the methods in Python’s scikit-learn library (version 1.2.2) [24, 25]. Spearman correlation test was determined by Python library Scipy [26]. Kaplan–Meier survival curves and log-rank test were drawn using the methods in Python’s library lifelines [27]. GSEA statistics and ssGSEA enrichment score were determined by GSEApy (version 1.1.1) [28]. Hazard ratio and Chi-square test were determined with GraphPad Prism (version 10.1.1). Spatial cell clustering was performed by Scanpy [29] and Squidpy [30]. The *p*-value below 0.05 and false discovery rate (FDR) below 0.25 were considered statistically significant.

## Results

### The training, validation, and testing process for the fivefold cross-validation process

WSIs of H&E-stained HGSOC tumor sections were downloaded from TCGA. The OS data for patients with stage III or IV cancer were used to train the model (target = 1 for patients with OS durations ≥ 36 months; target = 0 for patients with OS durations < 36 months). To obtain the accurate OS durations required for training, and to simplify the training process, uncensored patients from TCGA were selected for this study (773 images from 335 patients). The 773 images of the 335 patients were randomly split into a training and validation dataset for the fivefold cross-validation process and a testing dataset as described in methods. The metadata of the training, validating, and testing TCGA images are shown in Supplementary Table S1. To take into account the randomness of the training process, and to select the best possible model, 5 trainings were done for each fold, each fold with 10 epochs. The models with the best AUROCs, as evaluated with the validation datasets, were selected for each fold. The final H&E-based survival scores were calculated from the average scores of the selected 5 models from the 5 folds. The H&E-based survival scores, together with patient ages, were then evaluated using the TCGA training dataset, the TCGA testing dataset, and the MD Anderson Cancer Center testing dataset for prognosis prediction.

### The evaluation of the deep learning model using TCGA data

After training the model with the fivefold cross-validation process, the best AUROC values for each fold, which were obtained using validation images, were 0.705, 0.687, 0.628, 0.703, and 0.759. First, for the patients in the training/validating and testing dataset, we evaluated the effects of the ages at diagnosis and H&E-based survival scores on OS and PFS durations.

**Fig. 2:**
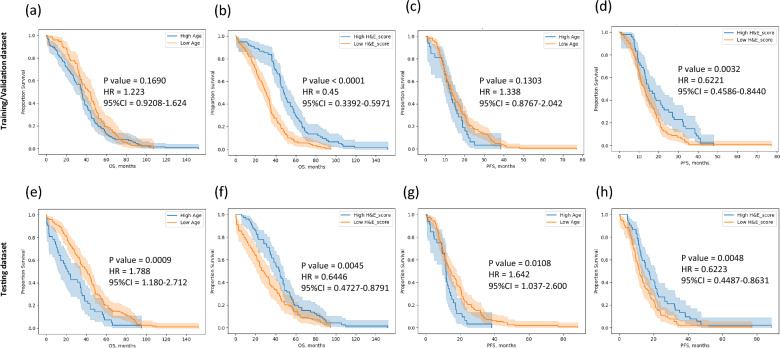
Kaplan–Meier curves demonstrating that the deep learning model was prognostic for OS and PFS durations in patients with HGSOC

The effects of the patients’ ages at diagnosis and H&E-based survival scores on survival were evaluated using Kaplan–Meier curves and the log-rank test (Fig. 2). For the patients in the training/validation dataset, the age at diagnosis was not prognostic for both OS and PFS duration (Fig. 2a, OS, *p*-value = 0.169; Fig. 2c, PFS, *p*-value = 0.1303), but it was prognostic for the patients in the testing dataset (Fig. 2e, OS, *p*-value = 0.0009; Fig. 2g, PFS, *p*-value = 0.0108). The H&E-based survival scores from the trained model were significantly prognostic for both the patients in the training and validation dataset (Fig. 2b, OS, *p*-value < 0.0001; Fig. 2d, PFS, *p*-value = 0.0032) and those in the testing dataset (Fig. 2f, OS, *p*-value = 0.0045; Fig. 2h, PFS, *p*-value = 0.0048).

We also evaluated if the model predicted patient prognosis based on other well-known prognostic covariate such as debulking status. We demonstrated that the AUROC of the model in predicting less optimal debulked (> 10 mm residual tumor) patients were 0.52 (data not shown). This suggests that the model did not predict patient prognosis based on debulking status.

The prognostic values of the age at diagnosis and H&E-based scores for OS and PFS duration were compared for patients with HGSOC. Kaplan–Meier curves and log-rank test results are shown for both the TCGA training/validation dataset (a, b, for OS; c, d, for PFS) and the testing dataset (e, f, for OS; g, h, for PFS). The panels show the results for the age at diagnosis (a, c, e, g) and the predicted H&E-based survival scores (b, d, f, h). Abbreviations: H&E, hematoxylin and eosin; HGSOC, advanced, high-grade, serous ovarian cancer; OS, overall survival; PFS, progression-free survival; TCGA, The Cancer Genome Atlas.

### Evaluation of the model using the MDACC dataset

The MDACC dataset, which consisted of 42 patients with HGSOC, was used for further evaluation of the deep learning model with patient characteristics shown in Supplementary Table S2. Images of H&E-stained patient tumors were scanned from H&E slides made from formalin-fixed, paraffin-embedded ovarian tumor blocks prepared from treatment naïve patients. As was done with the TCGA datasets, the MDACC dataset was used to evaluate the deep learning model. AUROC, Kaplan–Meier curves and log-rank tests were used to correlate the output scores and the patients’ 5-year OS durations.

**Fig. 3:**
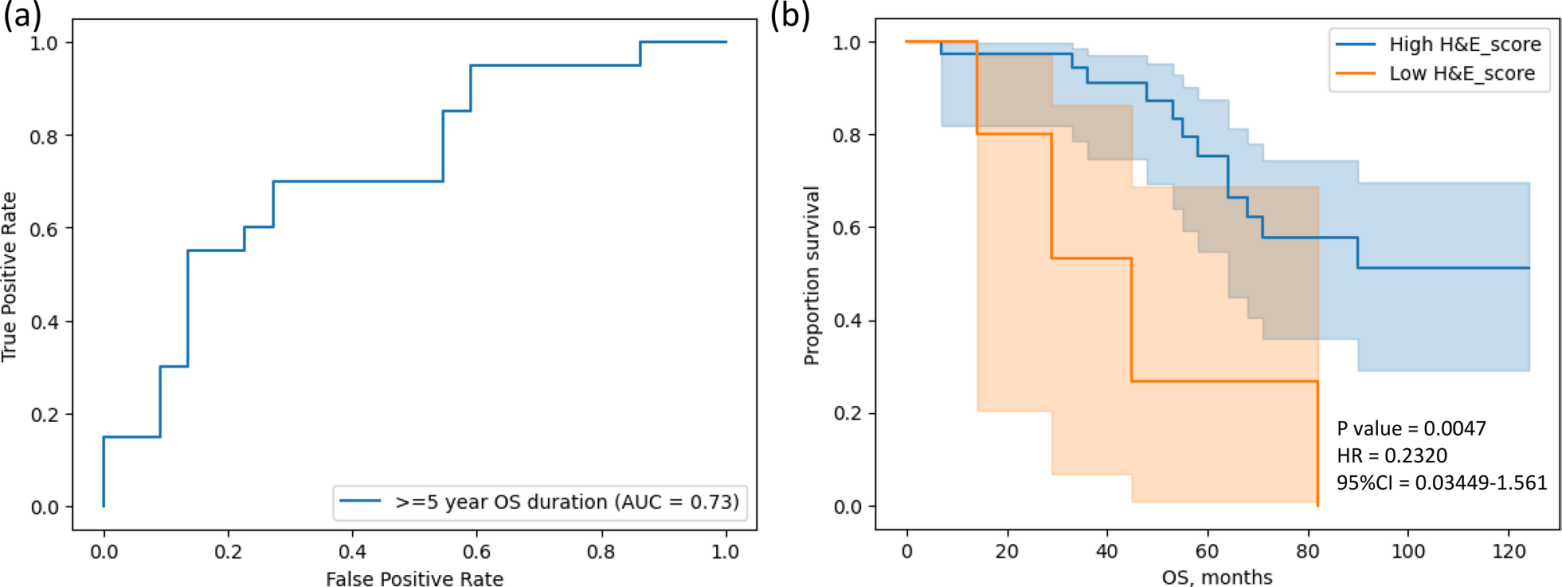
The evaluation of the deep learning model with the MDACC testing dataset

Using the AUROC method, we determined the performance of the model to predict patients with 5-year overall survival. The fivefold models had the AUROC results of 0.720, 0.686, 0.711, 0.736, and 0.707, and with AUROC 0.73 after averaging the 5 scores (Fig. 3a), which indicates that the model could predict the prognosis of the MDACC ovarian cancer patients. The Kaplan–Meier curve was shown (Fig. 3b) with the most significant log-rank test result (*p*-value = 0.0047) with a cut-off score of 0.448.

We also evaluated the performance of the model with or without stage I–II patients and similar results were obtained (data not shown), suggesting that the model did not predict prognosis based on stage information associated with the images.

Images of H&E-stained tumor sections obtained from the MDACC tumor bank were predicted for H&E-based survival score. The predicted H&E—based survival scores were evaluated for the 5-year survival prediction using AUROC (a) and OS durations using Kaplan–Meier curves and the log-rank test (b). Abbreviations: H&E, hematoxylin and eosin; MDACC, The University of Texas MD Anderson Cancer Center; OS, overall survival.

### Image features emphasized by the attention mechanism of the deep learning model

As described, the model trained on WSIs had an attention mechanism; such mechanism could improve the accuracy of the deep learning model, greatly enhancing its interpretability to help researchers better understand its decision-making and underlining mechanisms used to determine disease progression through both cancer cells and tumor microenvironment. However, the attention features learnt in pathological images are usually uninterpretable. We therefore employed spatial transcriptome to deconvolute the attention features.

**Fig. 4:**
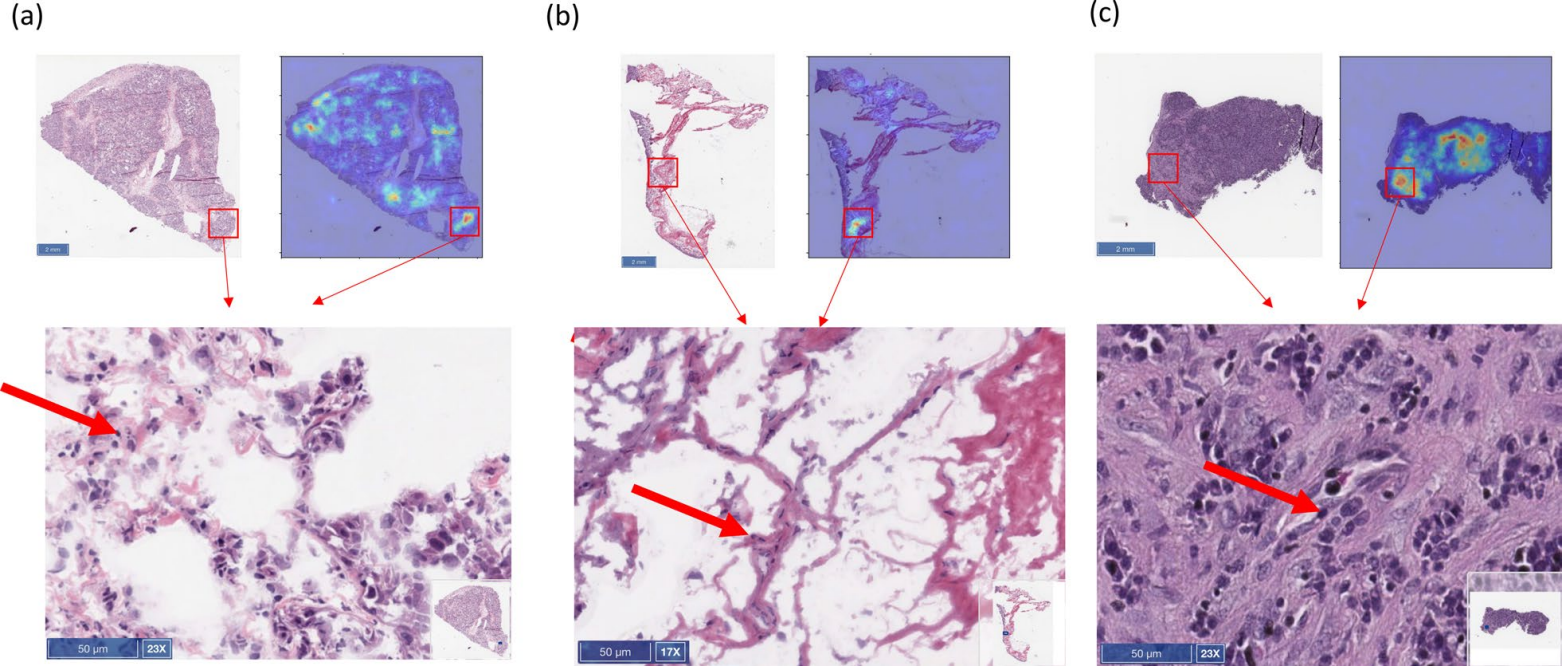
Image features emphasized by the attention mechanism of the deep learning model

The output of attention module 2 was extracted and overlaid with original images to form density maps. Red and blue coloring indicated regions with higher and lower importance, respectively, for the decision-making of the model (Fig. 4). Notably, the red regions fell mainly on the tumor tissue in both training and testing images instead of the blank areas. This indicated that the model had been trained well and performed its predictions using the features of the tumor region. Notably, immune infiltrates were seen in the area with high attention signal. We then interrogated the correlation between the attention signal and the immune signature.

The interpretability of the model was demonstrated by the intensity of the regions using the attention mechanism to examine the (a) TCGA training and validation, (b) TCGA testing, and (c) MDACC H&E-stained tumor images used in this study with red arrow highlighting immune cell infiltrations. Abbreviations: H&E, hematoxylin and eosin; MDACC, The University of Texas MD Anderson Cancer Center; TCGA, The Cancer Genome Atlas.

### Immuno-signature enrichment analysis reveals correlation between attention signal and immune activity

**Fig. 5:**
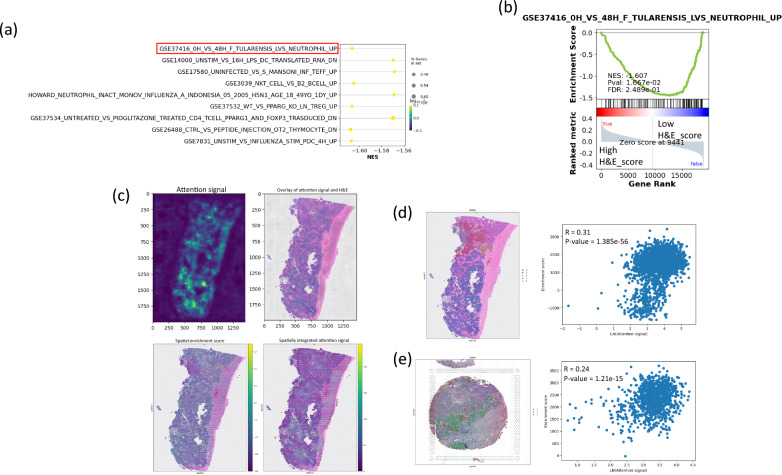
Immuno-signature enrichment analysis reveals correlation between attention signal and immune activity

To explore the underlying mechanism by which the signatures that the model learnt can predict the prognosis of the patient samples, we performed a pathway enrichment analysis to evaluate the differential immunological pathway activation between TCGA testing samples with low and high H&E—based survival score. By employing the gene expression data from cBioportal, GSEApy and Molecular Signature Database (MSigDB) [31–33], pathway enrichment analyses on testing TCGA dataset using the c7 immunological pathway gene set collection was performed. The samples were first labelled as high if their scores were higher than the median of the predicted H&E—based survival scores. The c7 immune signatures were then compared using GSEA test between the two groups, and the most significant signatures were selected for further analysis, and the results are shown in Supplementary Table S3. The enrichment scores of the 9 significant signatures were shown (Fig. 5a). The top signature GSE37416_0H_VS_48H_F_TULARENSIS_LVS_NEUTROPHIL_UP (Fig. 5b) was further validated for its relationship with the attention signal. The heatmap for the genes of the GSE37416_0H_VS_48H_F_TULARENSIS_LVS_NEUTROPHIL_UP immunological signature is shown in Supplementary Fig S2. Spatial transcriptome data of an ovarian cancer FFPE samples downloaded from 10X genomic were employed to investigate the relationship between the attention signal and immune signature. By using the enrichment scores for each spot in the samples as determined by the ssGSEA method, and integrating the attention signal detected from the whole H&E image of the spatial transcrioptome samples as spatial prognostic information of the samples (Fig. 5c), their correlation was determined by Spearman correlation test. We focused on the tumor cell cluster regions as these regions should have more prognostic information. Results showed that the attention signal in the tumor regions of the two ovarian cancer spatial transcriptome samples (Fig. 5d and e) were significantly correlated with the enrichment score (R = 0.31/0.24, P-value = 1.385e−56/1.21e−15). This suggests that the model predict the prognosis of the ovarian cancer patients by detecting specific types of immune activity.

The pathway enrichment analysis of the testing samples with low and high H&E—based survival score was done using GSEApy and c7 immunological signature gene sets (n = 5219) from MSigDB. The significant gene sets that were most significantly enriched are shown with the first 3 most significant signatures highlighted (a). The statistical results of the highlighted pathways GSE37416_0H_VS_48H_F_TULARENSIS_LVS_NEUTROPHIL_UP (b). The attention signal integration and GSVA enrichment score of the signature GSE37416_0H_VS_48H_F_TULARENSIS_LVS_NEUTROPHIL_UP of two spatial transcriptomic ovarian cancer FFPE samples were downloaded from 10X genomics. An example of attention signal and spatial transcriptome integration is shown (c), and the correlation of the enrichment score and the natural logarithm of attention signal of the tumor cell region the spatial FFPE sample (d, e) are shown.

## Discussion

The use of machine learning for applications such as cancer diagnosis and outcome prediction is growing in the field of pathology; however, the application of CNN models using H&E WSIs developed for outcome prediction in ovarian cancer patients has been limited due to their interpretability. There is an unmet urgent need in developing more efficient and interpretable prediction models for prognosis in patients with ovarian cancer. Such models would also form a base for the future research (e.g., the development of multimodality prediction models). In this study, we demonstrated that a machine-learning model trained, validated, and tested on H&E WSIs can predict survival in patients with HGSOC. The results of the model were found to be correlated with immuno-activity by integrating with spatial transcriptome analysis. This finding suggests that our model predicts clinical outcome with immunological information contained in images of H&E-stained tumor sections.

Deep learning model has been employed for learning the histopathological features associated with prognosis of cancer patients. However, the interpretability of deep learning is challenging especially when it is applied in clinical settings. In this study, to interpret the features that have been learnt by the model, an analytical method was performed to unravel the features the model trained to differentiate patient survival. As cell composition and cell signaling activity play crucial parts in cancer progression, and it is known that they can be learnt by deep learning model [15, 34], we tried to reverse this process by identifying the immunological signatures that are correlated with the predicted scores of the model. By this method, we identified immunological signatures that are related to the predicted histological survival scores.

Tumor-infiltrating lymphocytes have been found to be important in the prognosis of ovarian cancer patients. With histological images, ovarian cancer can be separated into groups with different risk by identifying TILs [35]. Different subtypes of TILs in ovarian cancer were also found to differentially affect the progression of ovarian cancer [36, 37]. From the pathway enrichment results in this study together with the spatial transcriptomes, we did a novel analysis to determine their relationship. We found that the histology-based output score of our model correlated with enriched immune signature associated with neutrophil [38], and other immune cell types as shown in Fig. 5a. Among them, the association with neutrophils is the most significant. The top gene in the enriched pathway associated with neutrophil is OASL, which has been shown to play a role in neutrophil recruitment..Studies also revealed its role in chemoresistance through T cell suppression [39–41]. Taken together, these findings suggest that certain immune features of the tumor sample could be learnt by the deep learning model to predict the prognosis of ovarian cancer patients.

One special aspect of the model described in this study is its simplicity. Via its attention mechanism, the model could select the most relevant tumor histology, including both the cancer cells and the tumor microenvironment, to make a prediction in an unbiased manner without being taught to look for a specific region of interest. As a result, this model predicted prognostic outcomes with minimal image processing. It is also noteworthy that, although the H&E images from the MDACC dataset and public spatial sample were inevitably slightly different from the images from the TCGA, this model generalized well when presented with images from different sources. This characteristic could allow the model to greatly reduce the technical barriers and costs of digital pathology.

Although this study has presented a model that may assist in predicting the prognosis of patients with HGSOC, it has several limitations. First, only uncensored patients were included. Since the model was trained using patients’ confirmed OS durations to simplify the model-training process, FocalLoss was used to calculate the resultant errors. While some would argue that this would lead to erroneous results due to the loss of information, the percentage of censored patients in the TCGA- ovarian cancer (OV Pan-Cancer) dataset was relatively low (37%), and the use of censored data could also potentially lead to biases due to the uncertain information within them [42]. Nevertheless, the samples of the MDACC dataset included both censored and uncensored data and showed significant results. Second, the majority of patients included in the training data were white, this means that the model could potentially perform better on white patients. Further investigation should be performed with dataset from patients with different ethnic background. Third, even though the attention mechanism highlights areas that might be linked to adaptive immune signatures, the correlation between those area and immune cell subtypes needs further investigation. Finally, the TCGA H&E images were split by images rather than patients in order to maximize the available images for training and testing.. Although this might result in information leakage, our evaluation results together with our own dataset and independent spatial transcriptome sample showed consistent results for both prognosis and pathway enrichment prediction.

In conclusion, we trained, validated, and tested a novel deep learning model with an attention mechanism using WSIs of H&E-stained tumor sections from patients with HGSOC. With the advancements of spatial omics platforms such as spatial transcriptomics [43], H&E-based predictive model can be integrated with these platforms to generate a prediction model with higher performance, and to provide insights into the morphological and immunological mechanism by which immunological features in tumor tissue link to the malignant phenotype of the disease. Further investigation into the clinical application of the model will need to be done by training and evaluating a full model with the whole dataset from more diverse patients.

## Supplementary Information

Additional file 1. Figure S1. Overview of image augmentation for training of the deep learning model. Rectangular images were converted into 2 JPEG files, 1 of which was flipped horizontally or vertically. Examples from 2 images are shown in. Images were normalized, vertically or horizontally flipped, and randomly affined before being fed into the model for training. Examples from 2 images are shown in. Figure S2. The gene heatmap of the top immune pathway of the pathway enrichment analysis. The gene heatmap of the most significant pathways between the predicted low and high H&E—based survival scores from the GSEA pathway enrichment analysis, which are GSE3039_ALPHAALPHA_CD8_TCELL_VS_B2_BCELL_UP, GSE14026_TH1_VS_TH17_UP, and GSE23114_PERITONEAL_CAVITY_B1A_BCELL_VS_SPLEEN_BCELL_IN_SLE2C1_MOUSE_UP are shown respectively.

Additional file 2. Table S1. TCGA images metadata for the 5-fold cross-validation process, training, and testing

Additional file 3. Table S2. The clinical characteristics of the patients included in the MDACC dataset

Additional file 4. Table S3. Immune signature enrichment results between TCGA testing samples with low and high H&E predicted score

## Data Availability

The TCGA data included in this study were downloaded from the GDC portal and cBioportal. The data from MDACC dataset are not publicly available due to patient privacy but are available upon reasonable request. The Jupyter notebooks used to generate the results is available as supplementary file.

## Abbreviations

FDR: False discovery rate
GDC: Genomic Data Commons
GSEA: Gene set enrichment analysis
H&E: Hematoxylin and eosin
HGSOC: High-grade serous cancer
MDACC: The University of Texas MD Anderson Cancer Center
MSigDB: Molecular signature database
OS: Overall survival
PFS: Progression-free survival
ssGSEA: Single-sample Gene Set Enrichment Analysis
TCGA: The Cancer Genome Atlas
WSI: Whole slide image
