# Supplementary figures and images for: Spatial transcriptome reveals histology-correlated immune signature learnt by deep learning attention mechanism on H&E-stained images for ovarian cancer prognosis
Source: J Transl Med. 2025 Jan 24;23:113. doi: 10.1186/s12967-024-06007-8 (PMC11761186; doi:10.1186/s12967-024-06007-8)

## Slide 1
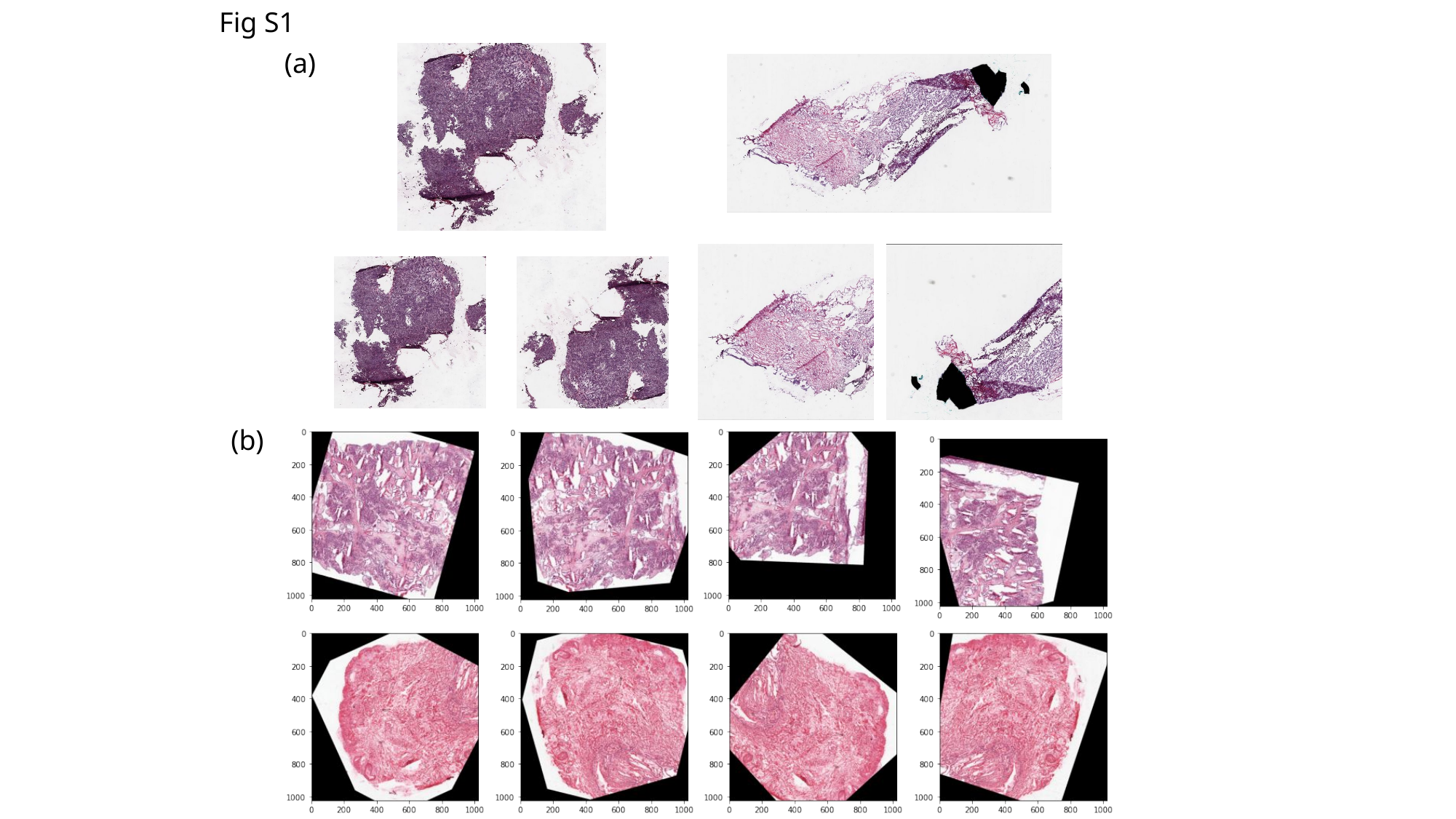

Fig S1
(a)
(b)

## Slide 2
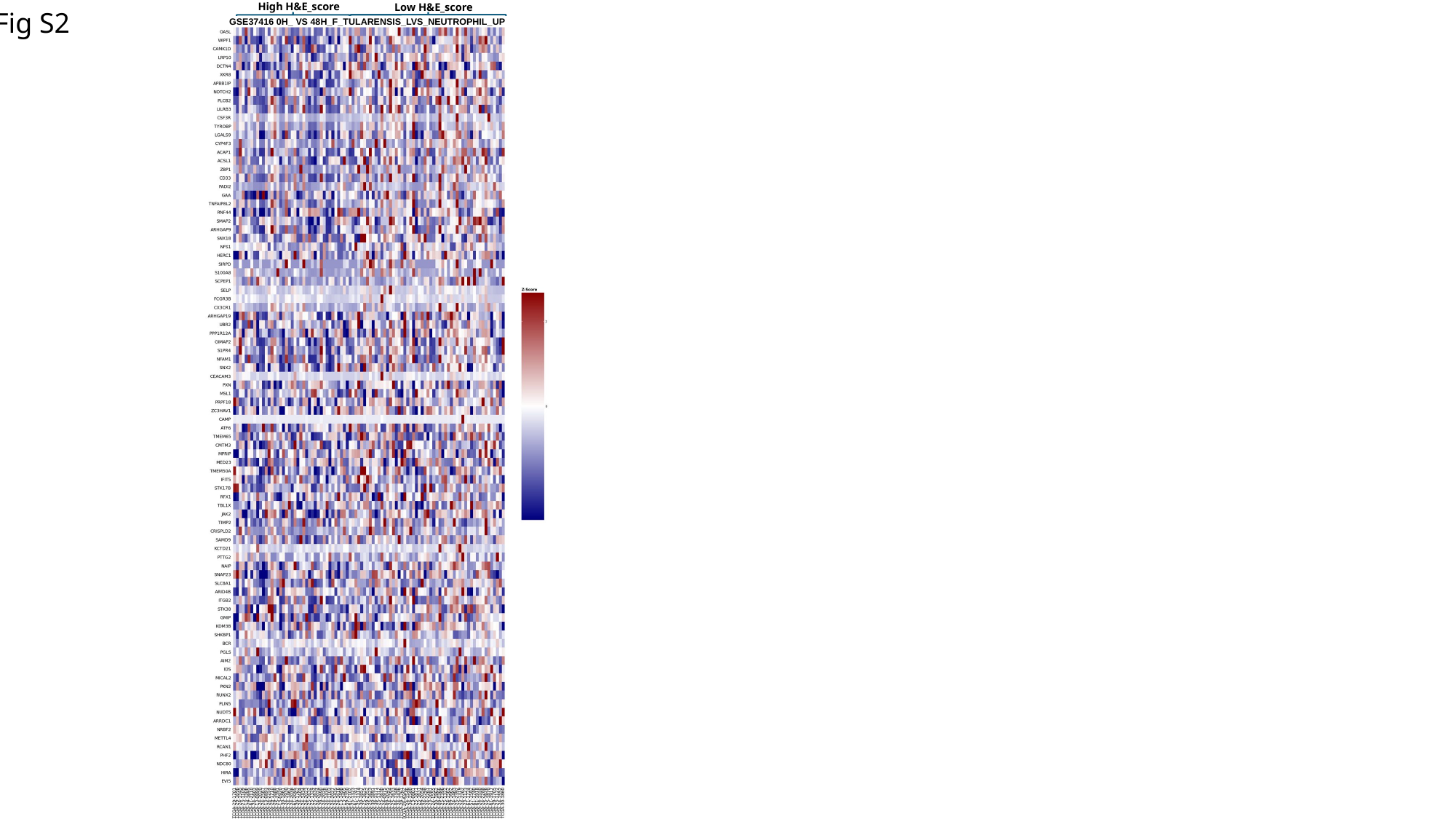

High H&E_score
Low H&E_score
Fig S2
GSE37416 0H_ VS 48H_F_TULARENSIS_LVS_NEUTROPHIL_UP

Supplement: Supplementary file 1 — Additional file 1. Figure S1. Overview of image augmentation for training of the deep learning model. Rectangular images were converted into 2 JPEG files, 1 of which was flipped horizontally or vertically. Examples from 2 images are shown in. Images were normalized, vertically or horizontally flipped, and randomly affined before being fed into the model for training. Examples from 2 images are shown in. Figure S2. The gene heatmap of the top immune pathway of the pathway enrichment analysis. The gene heatmap of the most significant pathways between the predicted low and high H&E—based survival scores from the GSEA pathway enrichment analysis, which are GSE3039_ALPHAALPHA_CD8_TCELL_VS_B2_BCELL_UP, GSE14026_TH1_VS_TH17_UP, and GSE23114_PERITONEAL_CAVITY_B1A_BCELL_VS_SPLEEN_BCELL_IN_SLE2C1_MOUSE_UP are shown respectively. [file 12967_2024_6007_MOESM1_ESM.pptx]
